# Supplementary material for: Soursop (Annona muricata) Properties and Perspectives for Integral Valorization
Source: Foods. 2023 Mar 29;12(7):1448. doi: 10.3390/foods12071448 (PMC10093693; doi:10.3390/foods12071448)
Supplement: Supplementary file 1 [file foods-12-01448-s001.zip › foods-2235603-supplementary.pdf]

Table S1

Soursop products available on the world market.

Soursop products	Country	Price range ($/100g)	Presentation
Dried leaves	German	10.00 – 900.00	Leaf 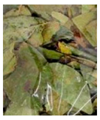
	USA		
	France		Ground 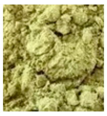
	Japan		
	Mexico		
	Sweden		Capsules 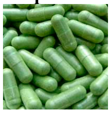
	Brazil		
Dried Pulp (soursop powder)	Brazil	3.00 – 130.00	Powder 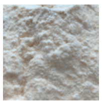
	Mexico		Capsules 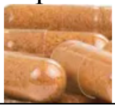
	Japan		
